# Suggestion without Sleep

**Published:** 1907-08-17

**Authors:** Douglas Bryan

**Affiliations:** Leicester


### Editor's Letter-Box.

### SUGGESTION WITHOUT SLEEP.

Sir,—I am rather surprised to see that Dr. Ash's letter, which is evidently intended to be a reply to mine, should commence with an entirely wrong statement. He begins by saying that my letter is a criticism of his article on "Treatment by Suggestion without Sleep." Now this is quite incorrect, for the only remark I made relating to the article as a whole was that I considered there were several debatable points in it; my remarks were solely made with reference to a statement by Dr. Ash on the condition of a hypnotised person. Practically speaking, he makes no reference to this except in a few lines in which he repeats his former misleading statement, though somewhat modified; I refer to the passage in which Dr. Ash suggests that I hypnotise a few willing people, etc. Nowhere in his letter does he attempt to explain or substantiate his original statement, but has evidently taken the opportunity to write further on the subject of suggestion without sleep, which is certainly recognised by all medical men practising hypnotism, and constantly used by them, though Dr. Ash, from the tone of his letter, would have us believe that little is known about it. I do not want, though, to take up this question at present until Dr. Ash has explained the points at issue; later I hope you will allow me space to criticise some remarks made by Dr. Ash in his last letter.

I am, faithfully yours,

Douglas Bryan.

Leicester, August 10, 1907.

